# Episiotomy and the risk of obstetric anal sphincter injury in nulliparous women with a prolonged second stage

**DOI:** 10.1007/s00404-026-08451-x

**Published:** 2026-04-29

**Authors:** Gal Bachar, Shahar Rosenthal, Nira Gridish, Naphtali Justman, Nizar Khatib, Ido Solt, Yaniv Zipori

**Affiliations:** 1 Ruth and Bruce Rappaport Faculty of Medicine, Technion-Israel Institute of Technology, Haifa, Israel; 2 Department of Obstetrics and Gynecology, Rambam Health Care Campus, 3109601 Haifa, Israel

**Keywords:** Episiotomy, Nulliparous, Obstetric anal sphincter injury, Second stage, Pregnancy

## Abstract

**Purpose:**

This study aimed to evaluate whether episiotomy reduces obstetric anal sphincter injuries (OASIS) rates in nulliparous women with a second stage of labor lasting ≥ 3 h.

**Methods:**

This retrospective study focused on nulliparous women at ≥ 36 weeks of gestation with singleton pregnancies who experienced a second stage of labor lasting ≥ 3 h and ultimately achieved spontaneous, non-operative, vaginal delivery between 2014 and 2024. Participants were categorized into two groups based on their episiotomy status. The primary outcome was the occurrence of OASIS, namely third- and fourth-degree perineal lacerations.

**Results:**

The study included 1164 (58.3%) women who underwent episiotomy and 831 (41.7%) who did not. Women in the episiotomy group were significantly younger (27.79 ± 4.31 vs. 28.47 ± 4.51 years, p < 0.001), had a higher prevalence of hypertensive disorders (7.7% vs. 5.2%, p = 0.029), experienced a slightly longer second stage of labor (3.62 ± 0.4 vs. 3.53 ± 0.4 h, p < 0.001), and delivered newborns with higher birthweight (3366 ± 390 vs. 3284 ± 376 g, p < 0.001). The OASIS rates were comparable between the groups (1.9% vs. 2.2%, p = 0.82), consistent across all subtypes and in a subanalysis of women with a second stage of ≥ 4 h (2.9% vs. 2.5%, p = 0.59). In adjusted multivariable analysis, episiotomy was not associated with OASIS (adjusted OR 0.95, 95% CI 0.48–1.84).

**Conclusion:**

In nulliparous women with spontaneous vaginal delivery and a prolonged second stage (≥ 3 h), episiotomy was not associated with a reduced risk of OASIS, even when the second stage exceeded 4 h. Our findings support existing guidelines that advocate against routine episiotomy in this population.

## What does this study add to the clinical work?

**Table:** 

This study provides robust evidence that a restrictive episiotomy policy remains justified and safe for nulliparous women, even within the context of a prolonged second stage (3–4 h). Our findings demonstrate that episiotomy does not reduce the risk of obstetric anal sphincter injuries (OASIS), which remains consistently low (under 3%) regardless of the intervention. Furthermore, avoiding episiotomy is associated with significantly lower postpartum pain levels and a higher likelihood of an intact perineum, without compromising neonatal or maternal outcomes. Ultimately, prolonged second-stage duration alone should not serve as an indication for episiotomy in modern obstetric practice	

## Introduction

A decade of contemporary labor data supports allowing up to 4 h in the second stage of labor for nulliparous women having regional anesthesia before diagnosing arrest [1–3]. However, several authors have questioned the safety of this approach, advocating for more cautious and less drastic changes in obstetric care [4–7]. In particular, a prolonged second stage increases the risk of obstetric anal sphincter injuries (OASIS), third- and fourth-degree perineal lacerations, which predispose patients to wound breakdown and infection. Women affected often experience lasting morbidities such as postpartum depression, dyspareunia, and pelvic floor disorders, including fecal and urinary incontinence [8–11]. OASIS not only poses serious clinical challenges but is also associated with substantial medicolegal implications and higher healthcare costs [12].

Current guidelines do not support the routine use of episiotomy in nulliparous women undergoing spontaneous vaginal delivery. A 2017 Cochrane review of eight randomized controlled trials (n = 5375) found that, in women anticipating an unassisted vaginal birth, a restrictive episiotomy policy was associated with a 30% reduction in severe perineal trauma (relative risk, 0.70; 95% CI 0.52–0.94) [13]. An updated 2024 systematic review confirms the evidence supporting a restrictive episiotomy approach [14]. However, the specific indications for performing this procedure remain undefined and are often based on individual judgment. Notably, none of the studies analyzed the duration of the second stage as an independent variable. The Cochrane review trials were conducted before the more prolonged second-stage duration, which typically limits it to 3 h in nulliparous women. A longer second stage may theoretically increase pelvic floor muscular tension, raising the risk of more extensive perineal trauma. In this higher-risk group, an episiotomy could mitigate OASIS risk.

The objective of this study was to evaluate the effectiveness of episiotomy, compared to no episiotomy, in preventing OASIS among nulliparous women having spontaneous vaginal delivery, within the context of a contemporary definition of prolonged second stage of labor.

## Materials and methods

### Study procedures

This retrospective cohort study was conducted at a tertiary referral center from January 2014 to January 2024. We provide care for approximately 15,000 patients annually, including nearly 5000 births, with nulliparous women accounting for a mean of 40% of deliveries. Comprehensive demographic, medical, and obstetric data, including intrapartum progression partogram, were obtained from the hospital’s electronic health record system. The study was approved by the Institutional Review Board, with a waiver of informed consent.

### Study protocol

Nulliparous women were eligible for analysis if they were at ≥ 37 weeks of gestation with a singleton pregnancy in vertex presentation, experienced a second stage of labor lasting ≥ 3 h (defined as a prolonged second stage), and achieved a spontaneous, non-operative vaginal delivery. Gestational age was based on the last menstrual period, confirmed by first-trimester ultrasound.

Participants were categorized into two groups based on their episiotomy status: (1) with episiotomy and (2) without episiotomy. Our labor ward practices a restrictive episiotomy policy, using the 60-degree mediolateral technique at the crowning of the fetal head when applied.

The primary outcome was the occurrence of OASIS in each group. OASIS was diagnosed at delivery by the attending obstetrician using Sultan’s classification [15]: grade 3a, involving < 50% of the external anal sphincter; grade 3b, involving > 50% of the external anal sphincter; grade 3c, involving complete disruption of the external anal sphincter with extension to the internal anal sphincter; and grade 4, involving both sphincters and the anorectal mucosa. In our department, OASIS is routinely repaired in the operating room under regional or general anesthesia by an on-call urogynecologist or colorectal surgeon. Secondary maternal outcomes comprised first- and second-degree perineal tears, postpartum hemorrhage (defined as estimated blood loss > 1000 mL), pain scores assessed by the visual analogue scale (VAS), analgesic requirements, breastfeeding rates, and hospital readmission within 6 weeks. Neonatal measures included umbilical artery pH < 7.0 and admission to the neonatal intensive care unit.

### Statistical analysis

Descriptive statistics for continuous variables are presented as mean ± standard deviation (SD). Categorical variables are expressed as frequency and percentage. Comparative analyses were conducted using the Student’s t-test or Mann–Whitney U test for continuous variables, depending on data distribution, and the chi-square test for categorical variables. A p-value < 0.05 was considered statistically significant. All statistical analyses were performed using IBM SPSS Statistics, version 28. Multivariable logistic regression was performed to assess the association between episiotomy and OASIS. Covariates were selected based on clinical relevance and statistical significance in univariate analysis, and included maternal age, hypertensive disorders, birthweight, and duration of the second stage of labor.

## Results

The study dataset comprised 1995 nulliparous pregnant women with a second stage duration ≥ 3 h, of whom 1164 (58.3%) underwent episiotomy and 831 (41.7%) did not.

**Fig. 1:**
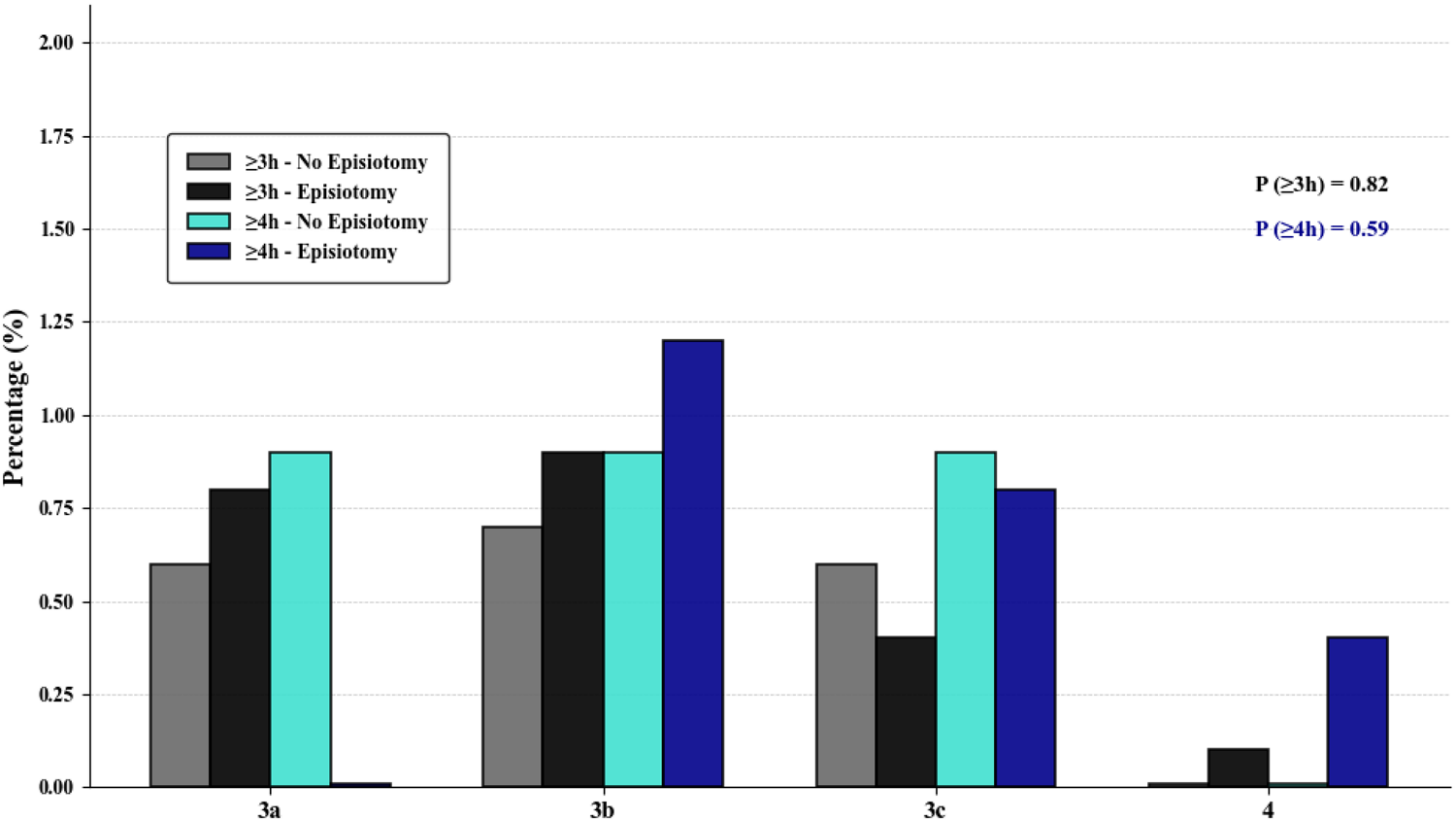
OASIS subtypes of episiotomy use and duration of second stage

Compared to the no-episiotomy group (Table 1), women in the episiotomy group were significantly younger (27.79 ± 4.31 vs. 28.47 ± 4.51 years, p < 0.001), had a higher prevalence of hypertensive disorders (7.7% vs. 5.2%, p = 0.029), had a slightly prolonged second stage of labor (3.62 ± 0.4 vs. 3.53 ± 0.4 h, p < 0.001), and delivered newborns with higher birthweights (3366 ± 390 vs. 3284 ± 376 g, p < 0.001). The difference in gestational age at delivery was clinically insignificant. Other intrapartum demographic characteristics were comparable between the two groups. The OASIS rates were similar between the groups (1.9% vs. 2.2%, p = 0.82), and Fig. 1 shows that these rates remained consistent across all OASIS subtypes. In a sub-analysis of women with a second stage of labor ≥ 4 h, 247 of 353 (70%) underwent episiotomy, with OASIS rates also comparable between the episiotomy and no-episiotomy groups (2.7% vs. 2.4%, p = 0.59). In multivariable analysis adjusting for maternal age, hypertensive disorders, birthweight, and duration of the second stage of labor, episiotomy was not associated with OASIS (adjusted OR 0.95, 95% CI 0.48–1.84). Table 2 presents the secondary maternal and neonatal outcomes observed in the two study groups. Women in the episiotomy group reported significantly greater postpartum pain during hospitalization (VAS score: 4.06 ± 3.2 vs. 3.61 ± 3.1, p = 0.003), with no significant difference in analgesic demand, including NSAIDs and paracetamol. By definition, all women undergoing episiotomy sustained a surgical second-degree perineal tear. In the no-episiotomy group, 32.6% sustained first and/or second-degree perineal tears (p < 0.001). Other maternal and neonatal outcomes, including breastfeeding initiation (92.7% vs. 91.9%, p = 0.55), did not differ significantly.

**Table 1:** Demographic characteristics stratified by the use of episiotomy

Maternal	No episiotomy N = 831	Episiotomy N = 1164	P-value
Maternal age, years, mean ± SD	28.47 ± 4.51	27.79 ± 4.31	<** 0**.**001**
Body mass index, kg/m^2^, mean ± SD	29.52 ± 4.76	29.28 ± 4.68	0.55
In vitro fertilization, n (%)	63 (7.6)	75 (6.4)	0.326
Gestational diabetes, n (%)	79 (9.5)	113 (9.7)	0.94
Hypertensive disorders, n (%)*	43 (5.2)	90 (7.7)	**0**.**029**
Regional anesthesia, n (%)	792 (95.3)	1121 (96.3)	0.30
PROM duration, h, mean ± SD	8.91 ± 5.88	8.97 ± 5.99	0.87
Use of oxytocin in labor, n (%)	724 (87.1)	1045 (89.9)	0.07
Chorioamnionitis, n (%)	77 (9.3)	116 (10.0)	0.65
Duration of second stage, h, mean ± SD	3.53 ± 0.38	3.62 ± 0.41	<** 0**.**001**
Gestational age at delivery, weeks, mean ± SD	39.55 ± 1.06	39.73 ± 1.07	<** 0**.**001**
Birthweight at delivery, g, mean ± SD	3283.94 ± 375.62	3366.04 ± 389.83	<** 0**.**001**

*n* number, *SD* standard deviation, *PROM* premature rupture of membranes

^*^Chronic, gestational, or preeclampsia

Values in bold indicate statistical significance (p < 0.05)

**Table 2:** Secondary maternal and neonatal outcomes stratified by the use of episiotomy

	No episiotomy N = 831	Episiotomy N = 1164	P-value
Maternal			
VAS score during hospitalization, mean ± SD	3.61 ± 3.1	4.06 ± 3.2	**0**.**003**
NSAID use, n (%)	166 (20)	232 (19.9)	1
Paracetamol use, n (%)	318 (38.3)	488 (41.9)	0.105
First- and second-degree laceration, n (%)	271 (32.6)	1164 (100)	<** 0**.**001**
RPOC, n (%)	96 (11.1)	160 (13.7)	0.154
PPH, n (%)	44 (5.3)	84 (7.2)	0.095
Need for blood transfusion, n (%)	21 (2.5)	35 (3.0)	0.58
Breastfeeding, n (%)	770 (92.7)	1070 (91.9)	0.55
Postpartum maternal hospitalization > 5 days, n (%)	33 (4.0)	46 (4.0)	1
Re-hospitalization within 6 weeks, n (%)	4 (0.5)	13 (1.1)	0.145
Neonatal			
Apgar score < 7 at 5 min, n (%)	3 (0.4)	6 (0.5)	0.74
Arterial cord pH < 7.0, n (%)	7 (0.8)	10 (0.9)	1
Admission to NICU, n (%)	17 (2)	24 (2.1)	1
Shoulder dystocia, n (%)	1 (0.1)	5 (0.4)	0.41
Perinatal mortality, n (%)	0	0	1

*n* number, *SD* standard deviation, *RPOC* retained products of conception, *PPH* postpartum hemorrhage, *VAS* visual analogue scale, *NSAID* nonsteroidal anti-inflammatory drugs, *NICU* neonatal intensive care unit

Values in bold indicate statistical significance (p < 0.05)

## Discussion

In nulliparous women with a prolonged second stage of labor and spontaneous vaginal delivery, there is a sharp increase in episiotomy rates at 3 h and surpasses 70% after 4 h. However, this intervention does not further reduce the risk of OASIS, which consistently remains below 3% irrespective of episiotomy use or OASIS subtype. Although episiotomy is associated with increased postpartum pain, it neither alters the need for analgesic demand nor related to other adverse secondary feto-maternal outcomes.

These findings are consistent with prior reports demonstrating that mediolateral episiotomy may not confer protection against severe perineal trauma and may even represent an independent risk factor for OASIS, even in critical conditions such as instrumental deliveries, occiput posterior position, and fetal macrosomia and non-reassuring fetal heart rate [16].

The episiotomy rate has dropped significantly in recent years, following RCTs that support a restrictive rather than routine approach to episiotomy in reducing OASIS [13, 17]. Despite the overall decline, overuse persists in specific geographic regions, particularly among nulliparous women, and in clinical scenarios such as impending severe perineal tears, shoulder dystocia, instrumental deliveries, non-reassuring fetal heart rates, and prolonged second stage of labor [18, 19]. Following the 2014 shift in obstetric practice that extended the duration of the second stage of labor and overall pushing time [1, 3], evidence regarding episiotomy use in this new context is limited. It remains unclear whether a restrictive episiotomy policy continues to offer protection against OASIS in nulliparous women under the updated guidelines. The present study addresses this gap, reporting the rate of episiotomy in nulliparous women experiencing a second stage lasting ≥ 3 h, including a subset whose second stage approaches the upper limit of 4 h. Regarding OASIS, a meta-analysis of seven studies by Pergialiotis et al. (n = 43,095) reported a significantly increased risk associated with a prolonged second stage (mean difference: 28.5; 95% CI 22.4–34.5), but the overall incidence remained low at 2.7%, a finding that is reassuringly consistent with our results despite an additional hour in the second stage [20].

In this study, women in the episiotomy group reported significantly higher average perineal pain than those in the no-episiotomy group, a finding consistent with existing literature indicating that more than half of women experience pain following an episiotomy [21]. Notably, there was no significant difference in analgesic demand between groups, suggesting that pain was adequately managed in our cohort. Given that suboptimal perineal pain control can adversely affect breastfeeding rates, mother-infant bonding, and the risk of postpartum depression [22, 23], our key finding, that avoiding episiotomy did not increase the risk of OASIS after a prolonged second stage, provides support for a restrictive episiotomy policy. In addition, prior studies have suggested that episiotomy does not improve perineal outcomes and may be associated with an increased risk of recurrent perineal trauma [24].

An episiotomy is a form of a controlled second-degree perineal tear, requiring similar postoperative care as spontaneous second-degree tears. Even after a prolonged second stage of labor, only one-third of women in the no-episiotomy group experienced first- or second-degree perineal tears, a rate that aligns with the wide range of 35–80% reported in the literature [25, 26]. Reassuringly, nearly 70% fewer women experienced any perineal tear, without an associated increase in the rate of OASIS.

### Clinical implications

The noticeable increase in episiotomy rates after 3 h, and even more so after 4 h, of the second stage of labor implies that labor duration itself is a significant factor affecting the procedure. However, since the data shows no corresponding decrease in OASIS, a restrictive episiotomy policy for spontaneous vaginal births remains justified, even when using a more permissive definition of a prolonged second stage.

RCTs are needed to establish clear indications for episiotomy during a prolonged second stage of labor. Furthermore, while this study focused on immediate OASIS outcomes, it underscores the need for research into the long-term sequelae of episiotomy use during prolonged labor, such as pelvic floor dysfunction, degrees of incontinence, and dyspareunia, given their potential to significantly impact quality of life.

### Strengths and limitations

This study presents a novel contribution to the understanding of episiotomy rates and their association with OASIS during second-stage labor that exceeds 3 h. The large cohort of nulliparous women, studied over 10 years under contemporary labor management guidelines, enhances the clinical applicability and external validity of the results. Furthermore, the application of multivariable logistic regression to adjust for confounders yields a more robust estimate of the independent impact of episiotomy on the risk of severe perineal injury. Notably, the absolute risk of OASIS remained low even with a prolonged second stage, underscoring the clinical relevance of these findings.

This study has several limitations, primarily due to its retrospective, single-center design.

First, the findings may not be generalizable to populations with different demographics, clinical protocols, or practices regarding episiotomy. Second, we could not account for known confounders influencing OASIS risk, such as perineal body length < 3 cm in nulliparous women [27, 28] and fetal occiput position [20], as these are not routinely documented in clinical practice; therefore, residual confounding by indication cannot be excluded despite multivariable adjustment. Lastly, the study did not evaluate long-term maternal outcomes of episiotomy in cases of prolonged second stages.

## Conclusions

In nulliparous women with spontaneous vaginal delivery with a prolonged second stage (≥ 3 h), episiotomy was not associated with a reduced risk of OASIS, even when the second stage exceeded 4 h. Although episiotomy use increased with longer second-stage durations, OASIS rates remained consistently low. These findings support existing guidelines advocating against routine episiotomy in this population. More research is needed to validate these results and to refine strategies to prevent OASIS during the second stage of labor.

## Data Availability

No datasets were generated or analysed during the current study.
